# Piezoresistive Behaviour of Additively Manufactured Multi-Walled Carbon Nanotube/Thermoplastic Polyurethane Nanocomposites

**DOI:** 10.3390/ma12162613

**Published:** 2019-08-16

**Authors:** Myoungsuk Kim, Jaebong Jung, Sungmook Jung, Young Hoon Moon, Dae-Hyeong Kim, Ji Hoon Kim

**Affiliations:** 1 School of Mechanical Engineering, Pusan National University, 2 Busandaehak-ro 63beon-gil, Busan 46241, Korea; 2 Division of Advanced Materials, Korea Research Institute of Chemical Technology, 141 Gajeongro, Daejeon 305-600, Korea; 3 School of Chemical and Biological Engineering, Institute of Chemical Process, Seoul National University, Seoul 08826, Korea

**Keywords:** 3D printing, fused deposition modelling, conductive polymer composite, piezoresistivity, 3D resistance network model

## Abstract

To develop highly sensitive flexible pressure sensors, the mechanical and piezoresistive properties of conductive thermoplastic materials produced via additive manufacturing technology were investigated. Multi-walled carbon nanotubes (MWCNTs) dispersed in thermoplastic polyurethane (TPU), which is flexible and pliable, were used to form filaments. Specimens of the MWCNT/TPU composite with various MWCNT concentrations were printed using fused deposition modelling. Uniaxial tensile tests were conducted, while the mechanical and piezoresistive properties of the MWCNT/TPU composites were measured. To predict the piezoresistive behaviour of the composites, a microscale 3D resistance network model was developed. In addition, a continuum piezoresistive model was proposed for large-scale simulations.

## 1. Introduction 

Conductive polymer composites are widely used in tactile, thermal, electrical, and chemical fields and are also used in electromagnetic interference (EMI) shielding and strain detection [1]. Conductive composites can be produced by injecting conductive fillers such as carbon black, carbon nanotube, graphene, or copper into a polymer matrix. If the distance between the fillers falls below a certain value, electrical conduction may occur through the fillers with the help of the tunnelling effect [2,3]. If sufficient conductive paths form with the increase in filler content, a percolation threshold is reached in which the electrical conductivity increases sharply. These conductive fillers can exhibit excellent conductivity even at low concentrations [4,5]. The minimum amount of conductive filler required to impart conductivity to the insulating polymers was previously calculated based on the percolation theory [6,7]. Recently, strain gauge sensors have been developed using conductive polymer composites. Yin et al. [8] developed a shear sensor to improve robot hand performance by collecting haptic information in real time. This sensor was produced as a transducer based on a liquid metal embedded in polydimethylsiloxane. Ruecha et al. [9] developed a new nanocomposite based on graphene/polyvinylpyrrolidone/polyaniline and used it to improve paper-based cholesterol biosensors. This biosensor exhibited significantly high electrochemical sensitivity for cholesterol detection owing to the high conductivity of the nanocomposites. Rein et al. [10] developed a buckypaper (BP) sensor to measure the strain of polymers having different elasticities. The BP sensor was fabricated by continuously filtering carbon nanotubes (CNTs) dispersed in a solvent. However, according to Sanli et al. [11], a strain sensor made of multi-walled carbon nanotubes (MWCNTs)/epoxy nanocomposites is better than the BP sensor because of the vulnerability of BP and the small range of strain measurement. The problem in this case is that the solubility of CNTs in an epoxy matrix is low, and CNTs cannot be easily dispersed. The applications of conductive nanocomposites are increasingc owing to the development of multifunctional sensors even though sensor production is costly and time-consuming. However, such materials are unsuitable for large strain detection because they exhibit insufficient flexibility and are susceptible to brittleness. Thus, developing highly flexible sensors that can be used to measure large strains in a target area is necessary.

The piezoresistivity of CNT polymer composites is attributed to the variation of conductive networks, tunnelling resistance change, and the piezoresistivity of CNTs [12]. Among them, the change of network morphology and the piezoresistivity of CNTs are expected to play a major role in the piezoresistivity of CNT polymer composites for large deformation. There have been efforts to understand the effect of the morphology of the filler on electrical and mechanical properties. Amjadi et al. [13,14] investigated morphological changes in AgNW networks during deformation and found that bottlenecks in the network affect the resistance change in particular with low filler concentrations. Duan et al. [15] investigated the conductive network morphology evolution of carbon black/thermoplastic polyurethane and olefin block copolymer composites by electron and optical microscopy and rheological measurements. They were able to change the morphology of conductive paths by changing the compounding sequence and annealing, resulting in the resistance change. Cai et al. [16] explained the piezoresistivity of CNT/PDMS composites with the breakage of interbundle junctions by deformation and described the resistance increase using a Weibull distribution. Dalmas et al. [17] used a 3D network model to investigate the electrical properties of CNT polymer composites and found that the increase of the fibre tortuosity decreases the average fibre gyration radius decreases leading to an increase of the percolation threshold. Jin et al. [18] conducted coarse-grained molecular statics simulations for studying the electrical properties of CNT thin films and found that the ratio of the mean projected CNT length over the film length is related to the hysteresis of resistance. Jomaa et al. [19] evaluated the effect of CNTs on the mechanical properties of CNT/PU composites by analysing the CNT network morphology and found that the CNTs do not stiffen the composite much because of the waviness, but increases the modulus significantly when stretched.

Three-dimensional (3D) printing, which is also known as additive manufacturing (AM) or rapid prototyping, can be used to fabricate layered 3D objects from 3D modelling data. Models can be produced in a very short time and can be modified quickly and cost-effectively, as moulds are not required in the production process. Furthermore, the design can be modified easily because it is based on 3D data. The AM methods can be subcategorised into stereo lithography apparatus, selective laser melting, selective laser sintering, laminated object manufacturing, and fused deposition modelling (FDM) [20,21]. In FDM, parts are fabricated by stacking layers through extrusion along different paths (X, Y, and Z axes) controlled by a computer in a semi-liquid state of the material melted by heating [22]. FDM is widely used because parts can be fabricated quickly at a low cost using various materials such as thermoplastic polymers, metals, and ceramics [23]. In addition, various functional filament materials such as conductive, photochromatic, fusible, flexible, and magnetic materials have been developed to produce functional parts using FDM [24,25,26]. The development of these functional filament materials is expanding the application of the FDM method. For example, a soft actuator, which is used for smooth manipulation of tissues in biomedicine, was printed with a silicon elastomer material [27], and fusible molds printed using a soluble polymer and a poly(vinyl alcohol) filament were used to produce complex artificial organs [28]. 

This study develops a piezoresistive nanocomposite using additive manufacturing with conductive fillers (i.e., MWCNTs) embedded in a very flexible and elastic thermoplastic polyurethane (TPU) matrix to improve electrical conductivity and piezoresistivity. Since the MWCNTs used in this study have a very large aspect ratio [29], if they are uniformly dispersed in the composite, forming a conductive path is easy. Thus, 3D printing is an effective method in terms of time and cost, as customized sensors can be fabricated without the need for complex deposition or assembly. To design highly sensitive pressure sensor materials, conductive composite samples with various MWCNT contents were fabricated, and the piezoresistive characteristics of MWCNT/TPU composites during loading were measured. To predict the piezoresistive behaviour of the composites, we developed a 3D resistance network model of the MWCNT/TPU composites, thus enabling the prediction of electrical conductivity and the representation of resistance variation in terms of the deformation of MWCNT/TPU composites. In addition, a continuum piezoresistivity model was proposed for large deformation, which could be used for macroscale simulations. 

## 2. Materials and Methods

### 2.1. Materials

CNTs are widely used as fillers for polymers owing to their nano-sized structure and excellent mechanical, physical, electrical, and thermal characteristics [30,31]. CNTs can be classified into three types: single-walled CNTs (SWCNTs), double-walled CNTs, and multi-walled CNTs (MWCNTs). The basic characteristics of SWCNTs are better than those of MWCNTs. However, SWCNTs are difficult to synthesize and are expensive. Therefore, in this study, we employed MWCNTs, which are widely used in low-cost applications owing to the ease of mass syntheses and commercialization. The MWCNTs used in this study had a purity of 90%, a diameter in the range of 8–15 μm, an aggregate size in the range of 5–100 μm, and a bundle length in the range of 10–80 μm. They were obtained in a powder form (Kumho Petrochemical Co., Ltd., Seoul, Korea).

TPU was used as a matrix material for the conductive composite, which is flexible, nonconductive, and 3D-printable. The Shore hardness of the TPU used in this study (ESTANE, Wickliffe, OH, USA) was 95 A. The TPU and CNTs were dried at 70 °C for at least 1 h prior to the mixing process. 

### 2.2. Fabrication of MWCNT/TPU Compounds and Filaments

**Figure 1:**
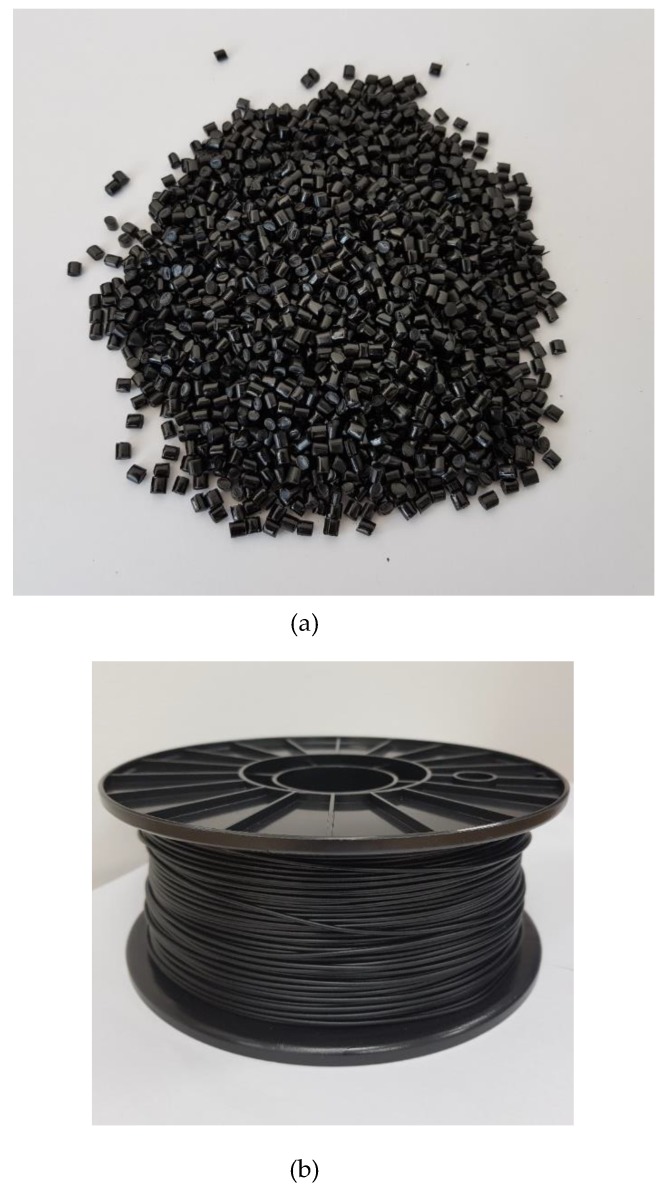
(**a**) Pellets and (**b**) filaments of the Multi-walled carbon nanotubes/thermoplastic polyurethane (MWCNT/TPU) composite.

**Figure 2:**
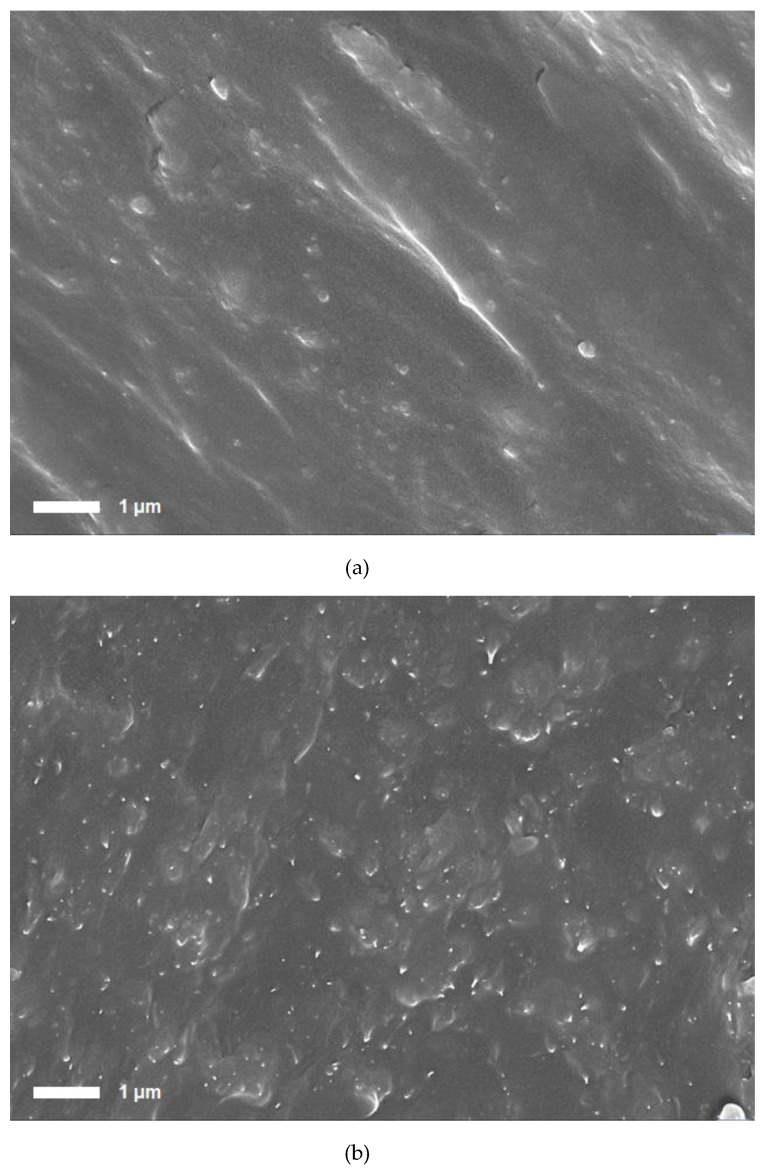
Scanning electron micrographs of (**a**) the pellet and (**b**) the filament of the MWCNT/TPU composite.

The MWCNT/TPU compounds were fabricated by extrusion (CNT Solution, Co., Ltd., Cheonan, Korea). First, the MWCNT and TPU were mixed in a blender at a high rotational speed. The mixed materials were then transferred to an extruder and were melted in a barrel heater. The MWCNTs were dispersed in the TPU matrix through the rotation of twin screws. The melting temperature was 200 °C, the length-to-diameter ratio of the twin screws was 45, and the rotation speed was 400 rpm. Since MWCNTs can easily coagulate and because the TPU is highly adhesive, a high shear force and heat must be applied to disperse the MWCNTs [32]. This is accomplished by rotating the twin screws at a high speed. The water-cooled MWCNT/TPU composites were extruded in strands and transferred to a pelletizer to cut them into pellets using air pressure while cooling them in water (Figure 1a). The pellets were moved to a water tank and were dehydrated by rotating the centrifuge chamber at a high speed. Thus, 3D-printable filaments (Figure 1b) were produced by extruding the pelletized MWCNT/TPU composites through the extruder (JPM Co., Ltd., Busan, Korea). The diameter of the extruder nozzle for filament production was set to 2.8 mm and the extrusion temperature to 200 °C. The filaments produced through the extrusion process were cooled and contracted while passing through the water tank and were wound around a spool. To use them in a 3D printer, the standard deviation of the filament diameter must be low and constant. To obtain filaments with a fixed diameter, the screws were rotated at a speed in the range of 55–70 rpm. The resulting diameter of the MWCNT/TPU filaments was measured to be 1.65 mm with a standard deviation of 0.05 mm. To examine the piezoresistivity, four types of filaments with different MWCNT concentrations (2, 3, 4, and 5 wt%) were prepared. To examine the homogeneity of the dispersion of the MWCNT, the scanning electron micrographs of the pellet and the filament were taken, as shown in Figure 2. Though CNTs in the pellet were not clearly identifiable, it was found that CNTs are well dispersed in the filament. It was not observed whether the CNTs broke during the processing from the micrographs. It was reported, however, that the morphology of CNTs remain almost unchanged after multiple extrusions [33].

### 2.3. 3D Printing 

**Figure 3:**
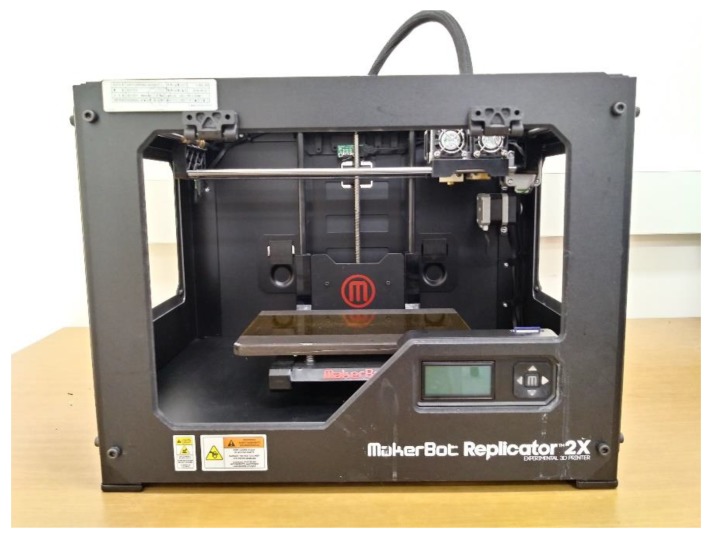
Fused deposition modelling (FDM) 3D printer.

The samples used in the experiments were printed using a commercial FDM 3D printer (Makerbot replicator 2X, MakerBot Industries, Brooklyn, NY, USA), as shown in Figure 3. Because of the characteristics of the MWCNTs, they can coagulate in the nozzle during the 3D printing process. Moreover, the print quality becomes poor due to the uneven extrusion of the filaments or the clogging of the nozzle [34,35]. If the nozzle is clogged, the MWCNT/TPU filaments are twisted in the extruder, which can damage the 3D printer. To control the coagulation of the MWCNT fillers, a TPU with a relatively high hardness (Shore hardness: 95 A) was selected, and a nozzle diameter of 0.8 mm was used. In addition, the extruder temperature of 230 °C was used, which was higher than that used for pure TPU (210 °C). 

A high printing speed of 85 mm/s was used to minimize the residence time during the melting of the MWCNT/TPU filaments at the hot end. Table 1 lists the printing conditions for MWCNT/TPU specimens used in this study. 

**Figure 4:**
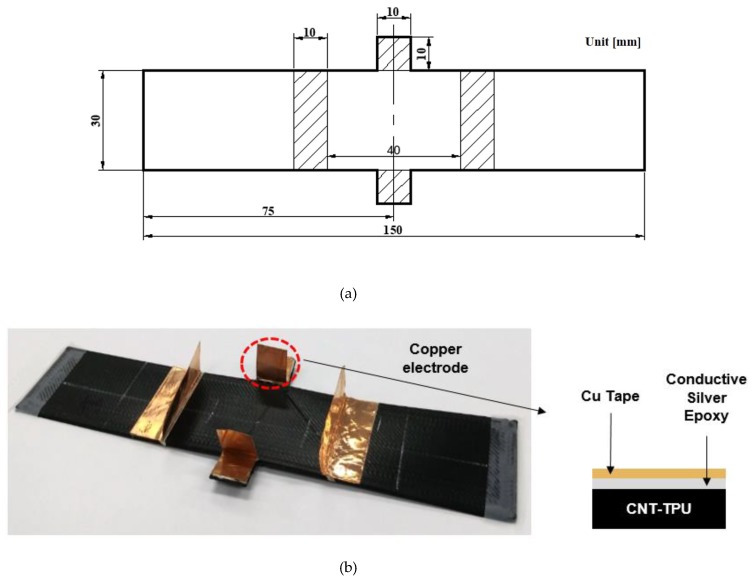
(**a**) Dimensions and (**b**) copper electrodes of the specimens for tensile tests.

We fabricated specimens for the tensile test using an in-situ measurement of piezoresistivity characteristics, as shown in Figure 4a. The thickness of the specimens was 1.2 mm. The infill percentage was 100%, and the infill angles were +45°/−45° to the longitudinal direction of the specimen. Square-shaped areas (10 mm × 10 mm) were added on both sides of the specimen at its centre to measure the resistance along the transverse direction during tensile tests. Finite element (FE) simulations confirmed that the effect of the addendums on the mechanical behaviour of the specimen was minimal. Copper electrodes were attached to the specimens using silver epoxy for measuring resistance along the longitudinal and transverse directions, as shown in Figure 4b. The contact resistance between the specimen and copper tape was reduced by sufficiently drying the specimens for approximately one day. To minimize the effect of interfaces between the printed layers and residual stresses generated during the printing process and thus to obtain reliable resistance data, the specimens were heat-treated in a heating chamber at 180 °C for 10 min, which was lower than the melting point of the TPU (210 °C).

### 2.4. Characterization 

To compare the initial conductive characteristics of the specimens with variations in MWCNT concentration, the resistances of the specimens fabricated using the 3D printer were measured. The resistance was then measured for 1 min using a multi-meter (Data Acquisition/Switch Unit, Keysight Technologies Inc., Santa Rosa, CA, USA) connected to the copper electrodes after a stable signal was obtained. The conductivity was calculated using the measured resistance and specimen geometry. 

To obtain the stress–strain and piezoresistive behaviour of the MWCNT/TPU specimens, tensile tests were conducted using a universal testing machine while measuring the resistances between the electrodes. Strains were measured using a laser extensometer with a gauge length of 30 mm. The cross-head speed was 0.33 mm/s, which corresponds to a strain rate of 3.7 × 10^−3^ /s. To check the piezoresistivity during deformation, the variation of resistance was measured using the multi-meter while a loading test was performed along the longitudinal and transverse directions. 

### 2.5. 3D Resistance Network Model 

**Figure 5:**
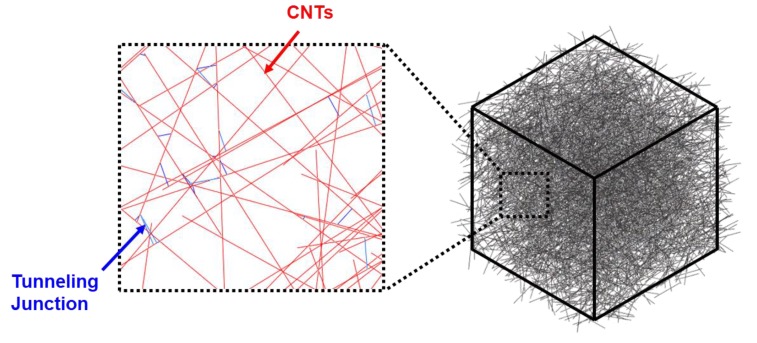
3D resistance network model.

A 3D resistance network model was developed to simulate the conduction phenomenon of the MWCNT/TPU composites using an in-house Fortran program and the commercial finite element program ABAQUS/Standard (Dassault Systèmes Simulia Corp., Johnson, RI, USA). A cubic unit cell of the size of 1000 × 1000 × 1000 nm^3^ was considered when CNTs were dispersed (Figure 5). The CNTs were represented as randomly oriented straight conductors or one-dimensional finite elements defined by two nodes. It was assumed that if the distance between the CNTs became less than the diameter, contact conduction occurred between the two conductors by the tunnelling effect. In addition, CNTs follow the global deformation of the unit cell at a fraction of α. Therefore, the resistivity of the CNT itself increases during deformation. 

In the finite element calculation, the conduction between the CNTs was accounted for by connecting the CNTs with another one-dimensional finite element. To calculate the resistance of the unit cells, the current was measured at a given voltage difference between the two sides of the unit cell. Initially, CNTs are randomly dispersed in a cubic unit cell. As the unit cell deforms, CNTs follow the macroscopic deformation of the unit cell. Linear elements with two nodes were used and the analysis was performed under steady state conditions.

### 2.6. Continuum Resistivity Model

The evolution of resistivity of the MWCNT/TPU composite was modelled in a continuum sense. The Ohm’s law is given by:

(1) E=ρj

where E and j are the field strength and current density, respectively. The resistivity tensor ρ may be expressed by:

(2) $$\rho =\rho \left[\begin{array}{ccc} 1 & 0 & 0\\ 0 & 1 & 0\\ 0 & 0 & 1 \end{array}\right]$$

where ρ is a resistivity parameter that depends on deformation. Here, the resistivity tensor is assumed to be isotropic. The ρ parameter may be expressed as:

(3) $$\rho =\rho_{0}\left(1+C\left(I_{1}-3\right)\right)$$

where $\rho_{0}$ is the resistivity of the un-deformed state, C is the material parameter, and $I_{1}$ is the first invariant of the stretch tensor, given by:

(4) $$I_{1}=\lambda_{1}^{2}+\lambda_{2}^{2}+\lambda_{3}^{2}$$

where $\lambda_{1}$, $\lambda_{2}$, and $\lambda_{3}$ are the three principal stretches. 

## 3. Results and Discussion

### 3.1. Mechanical Behaviour

**Figure 6:**
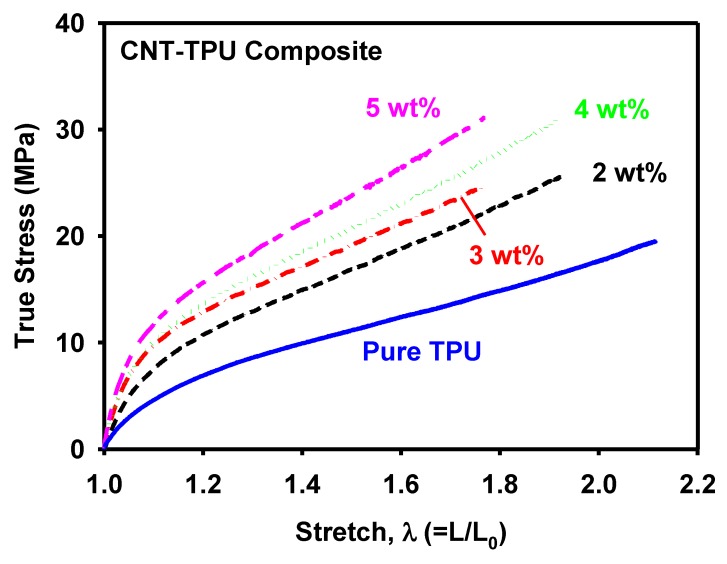
True stress-stretch curves of the MWCNT/TPU composites.

**Figure 7:**
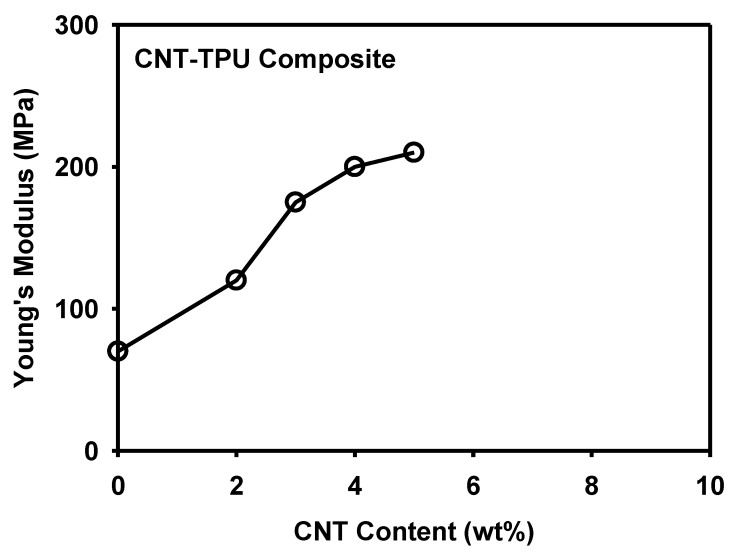
Young’s modulus of the 3D-printed MWCNT/TPU composites.

Figure 6 shows the true stress–stretch curves of the 3D-printed MWCNT/TPU composite specimens with CNT contents of 2, 3, 4, and 5 wt%. For comparison, a pure TPU specimen was also printed and tested. It was confirmed that the strength of the specimen increased with the concentration of the MWCNT conductive filler, which was in agreement with previous reports [36,37]. The Young’s modulus was determined from the stress–strain curve in the initial 5% strain range, as shown in Figure 7.

### 3.2. Electrical and Piezoresistive Behaviour

**Figure 8:**
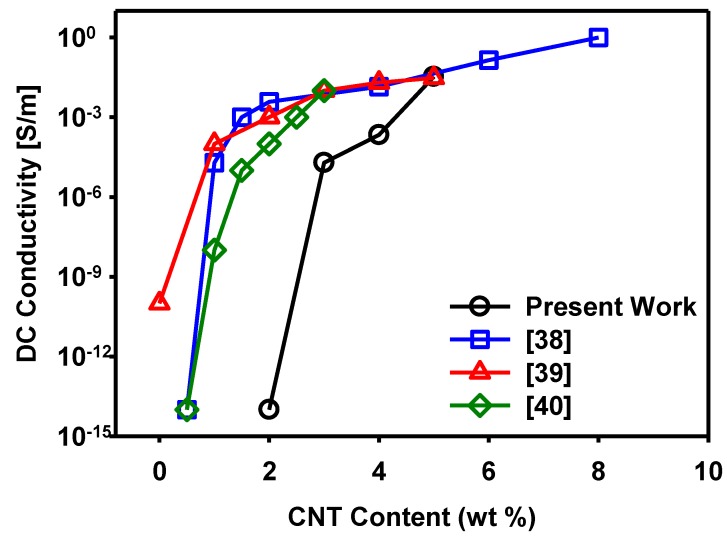
Variation of electrical conductivity with respect to the carbon nanotubes (CNT) content.

Figure 8 shows the initial electrical conductivity of the 3D-printed specimens of the MWCNT concentrations of 3%, 4%, and 5%. With the increase in MWCNT content, the conductivity of the MWCNT/TPU composites increased. Notably, the conductivity increased sharply at an MWCNT concentration of 5 wt%, which could be regarded as the percolation threshold. In the previous report, we observed that the conductivity increased rapidly in the vicinity of the percolation thresholds and saturated slowly thereafter [38,39,40]. With the MWCNT concentration of 5 wt%, the conductivity is high, suggesting that a stable conductive network is formed. By contrast, no electrical conductivity was measured in the composite with an MWCNT concentration of 2 wt%. This suggests that an MWCNT concentration of 2 wt% is insufficient to form stable conductive networks in the TPU matrix. 

**Figure 9:**
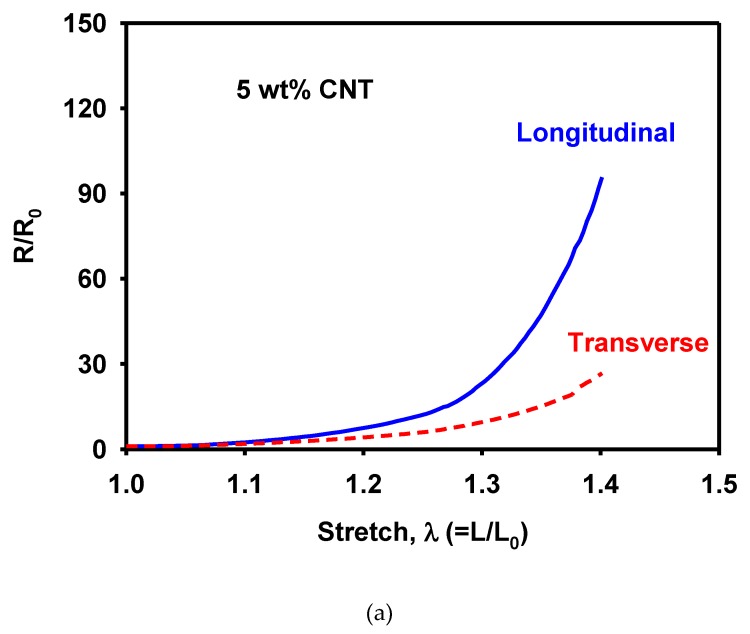

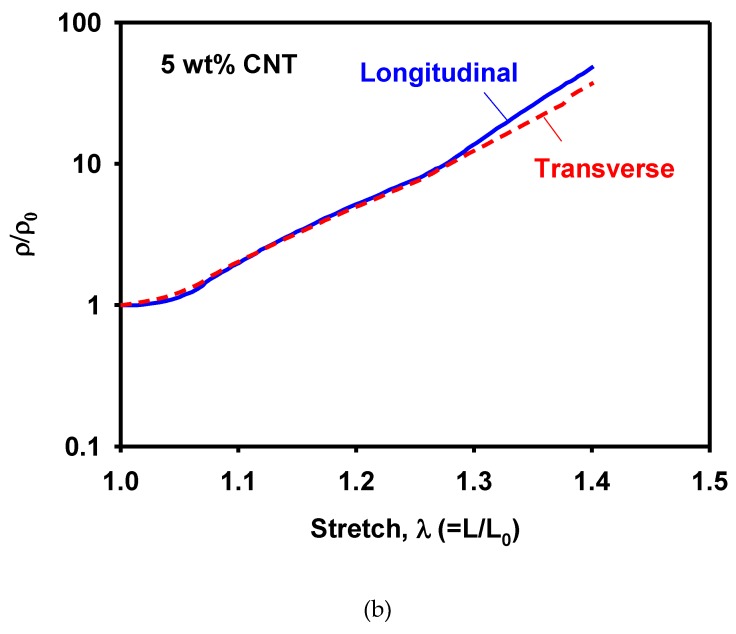
Measured variation of (**a**) normalized resistance and (**b**) normalized resistivity with respect to stretch during uniaxial tension.

The piezoresistivity was measured using resistance variation under loading. The MWCNT/TPU composites with a MWCNT concentration of 5 wt% were used. The resistance was measured under a tension up to a stretch of 1.4. Figure 9a shows the normalized resistance in terms of the stretch. The resistance increased at a faster rate in the longitudinal direction than in the transverse direction. The resistance data had the geometrical effect of deformation: the length between the electrodes increased in the longitudinal direction, whereas that in the transverse direction decreased during uniaxial tension. The normalized resistivities in the longitudinal and transverse directions agreed with each other, as shown in Figure 9b, implying that the isotropy of resistivity was preserved during deformation. 

**Figure 10:**
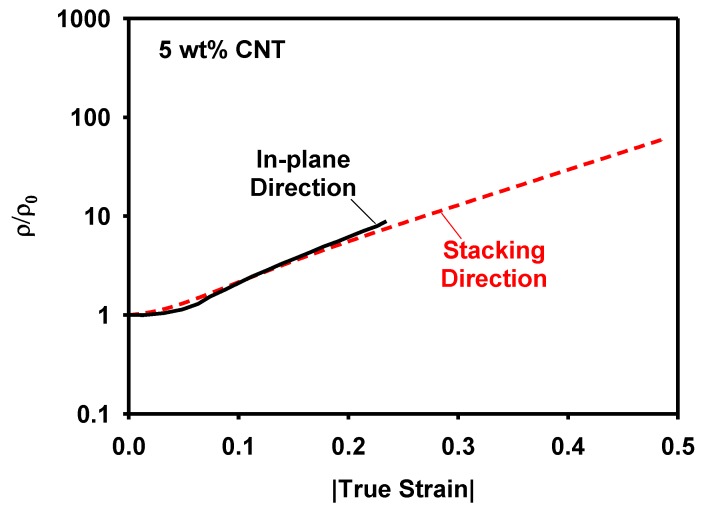
Comparison of the normalized resistivity in the in-plane and the stacking directions.

In order to evaluate the anisotropy of the piezoresistivity, possibly inherited from the printing process, the resistivities in the in-plane and stacking directions were compared in Figure 10. The piezoresistivity in the stacking direction agrees well with that in the in-plane direction.

**Figure 11:**
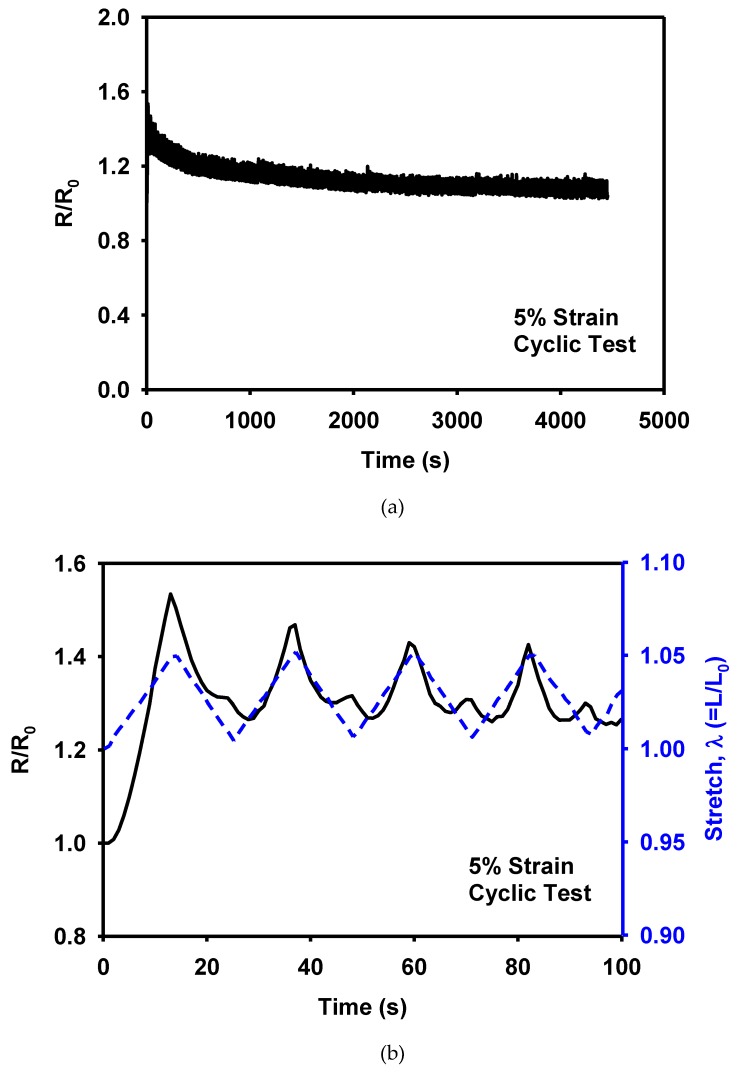

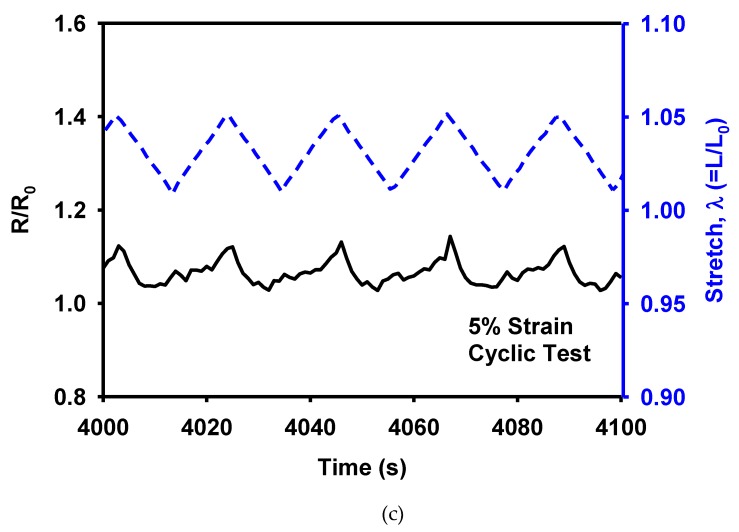
Evolution of piezoresistivity of the MWCNT/TPU composite during cyclic deformation: (**a**) the entire 200 cycles, (**b**) the initial cycles, and (**c**) the cycles after 180 cycles.

In order to demonstrate the reliability of the 3D printed MWCNT/TPU composites, the piezoresistivity was measured for cyclic deformation. Figure 11 shows the normalized resistance during 200 cycles in the strain range from 0% to 5%. The peak of the resistance decreases with cycles, and small peaks appear at the lower limit of strain. The observed behaviour is in accordance with the previous reports [16,41,42]. The decrease of the peak resistance may be attributed to the stabilization of the network morphology. The appearance of small peaks in the resistance is explained by the buckling of the CNTs [18,43]. 

The variation in the resistivity during uniaxial tension was calculated by the 3D resistance network model. Material parameters are listed in Table 2. In this work, the aspect ratio or the ratio of length to diameter of CNTs was limited to 80 in the 3D resistance network model due to the difficulties in computation. 

**Figure 12:**
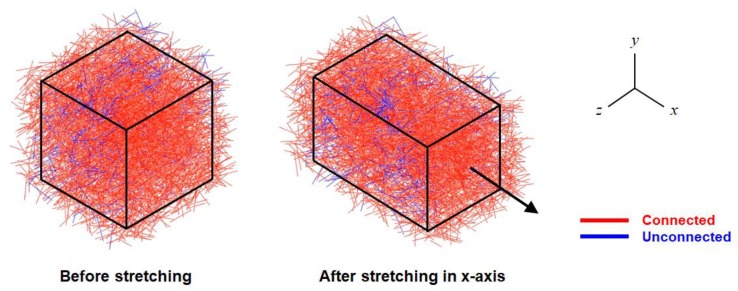
3D resistance network models before and after uniaxial tension in the x-axis at a stretch of 1.4.

**Figure 13:**
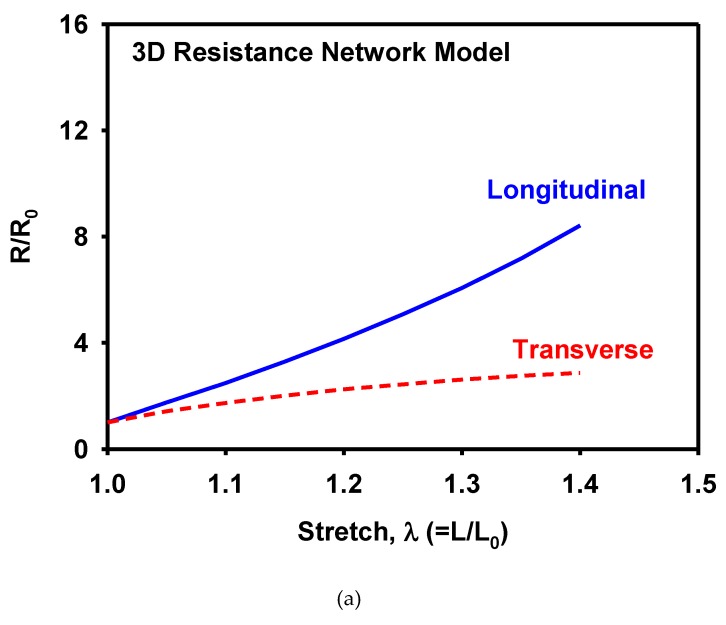

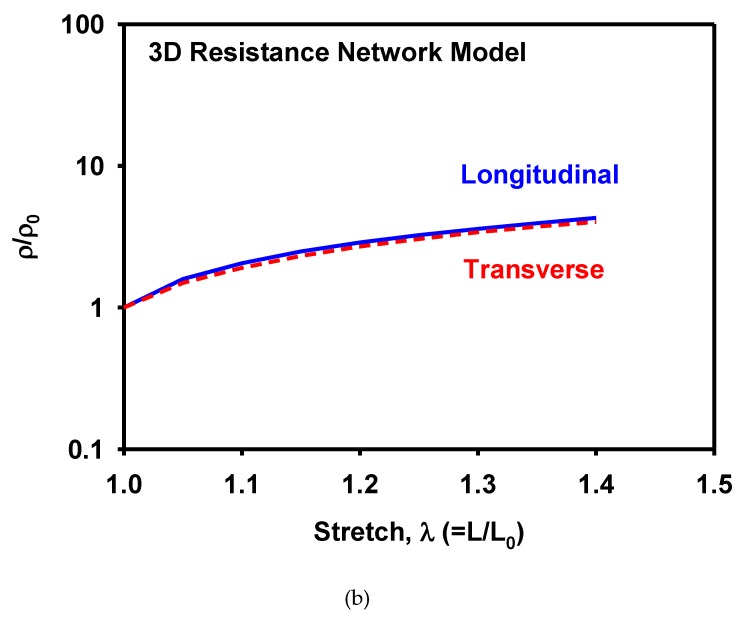
Variation of (**a**) normalized resistance, and (**b**) normalized resistivity with respect to stretch as calculated by the 3D resistance network model.

Figure 12 shows the 3D network model before and after the uniaxial tension corresponding to a stretch of 1.4 with colors representing connectivity. Figure 13a,b show the variations in normalized resistivity during uniaxial tension. Similarly, to the measurements (Figure 9b), the calculated resistivities in the longitudinal and the transverse directions showed little difference with each other, confirming isotropic evolution of resistivity. However, the shape of the resistivity curve showed the opposite trends: the measured curve is concave-up, whereas the predicted one is concave-down. It can be explained by the assumption of the straight CNTs used in the 3D resistance network model. In the CNT/TPU composite, CNTs have extremely large aspect ratios and are therefore entangled naturally regardless of their high stiffness. Upon stretching of the composite, the CNTs are straightened but they experience little stretching and thus little increase of resistivity until they are fully stretched. Therefore, the measured resistivity showed little change in the early stage of stretching. In the 3D resistance model, on the other hand, the already straight CNTs stretch proportionally with the composite, showing a large increase of resistivity from the beginning of stretching.

**Figure 14:**
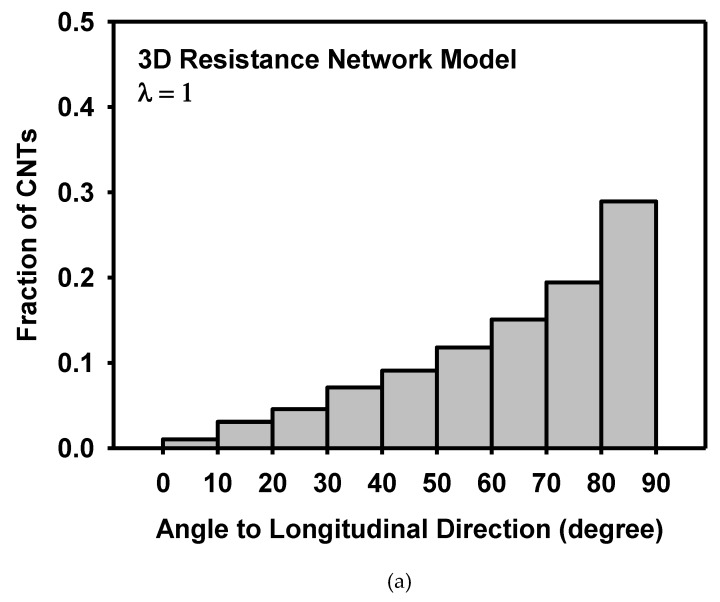

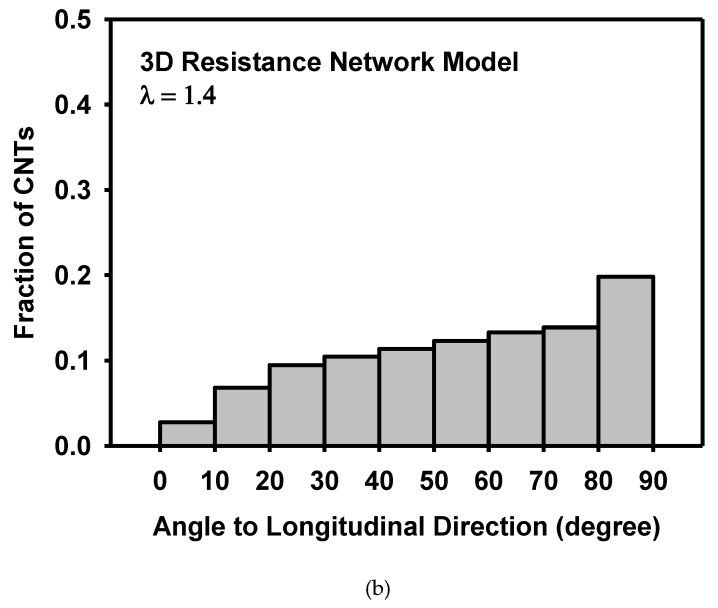
Distribution of the CNT angle to the longitudinal direction (**a**) at the initial state, and (**b**) at the stretch of 1.4 as calculated by the 3D resistance network model.

**Figure 15:**
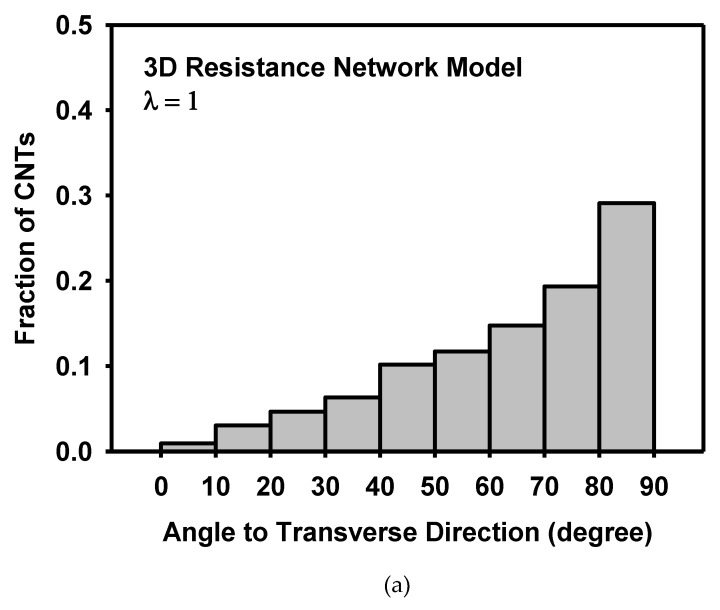

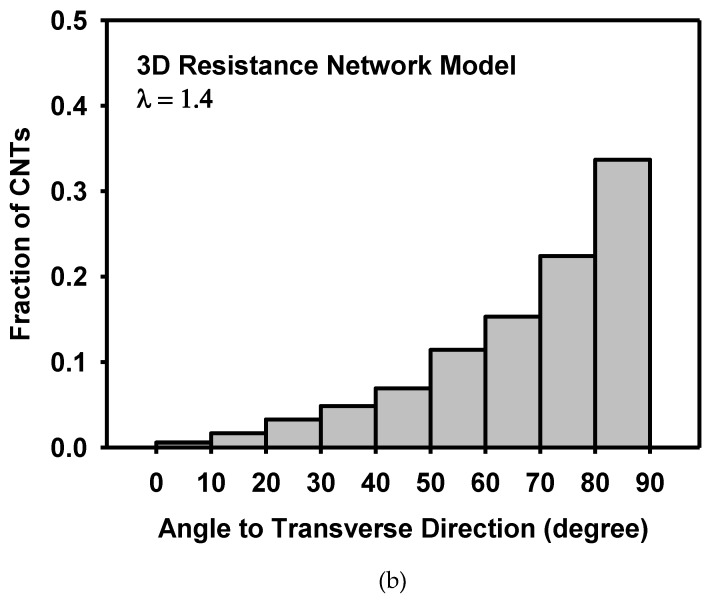
Distribution of the CNT angle to the transverse direction (**a**) at the initial state, and (**b**) at the stretch of 1.4 as calculated by the 3D resistance network model.

The morphological change of the resistance network during deformation is analysed as follows. Figure 14 and Figure 15 show the distribution of the angle of CNTs to the longitudinal and transverse directions, respectively. Initially, both CNT angles to the longitudinal and transverse directions showed random distribution. As the model is stretched in the longitudinal direction, CNTs with low angles to the longitudinal direction increases, implying that more CNTs are aligned to the longitudinal direction. On the other hand, CNTs with high angles to the transverse direction increases, indicating that CNTs are rotated away from the transverse direction. The anisotropic distribution of CNTs was caused by the deformation.

**Figure 16:**
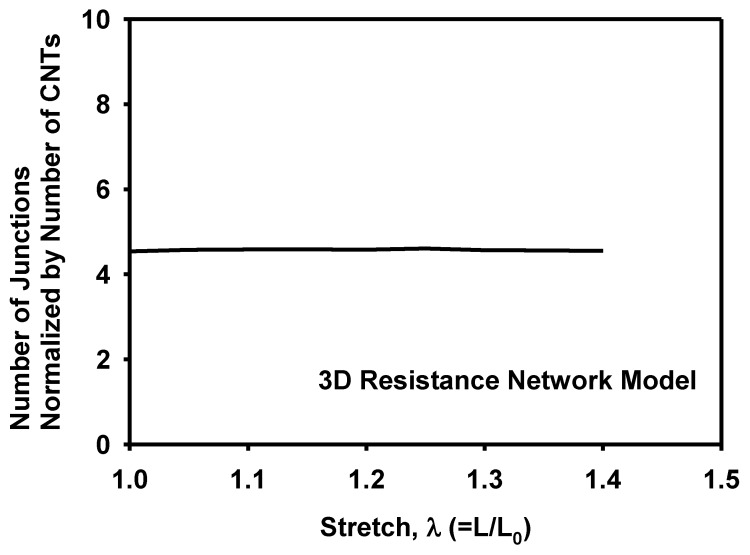
Evolution of normalized number of junctions between CNTs during stretching as calculated by the 3D resistance network model.

The number of junctions between CNTs did not change much during deformation, as shown in Figure 16. It does not necessarily mean that the junctions formed initially maintained during stretching. Instead, newly formed junctions compensate junctions destroyed during deformation. Overall, the number of junctions remain almost unchanged and therefore junctions did not affect the piezoresistivity of the composite much. 

**Figure 17:**
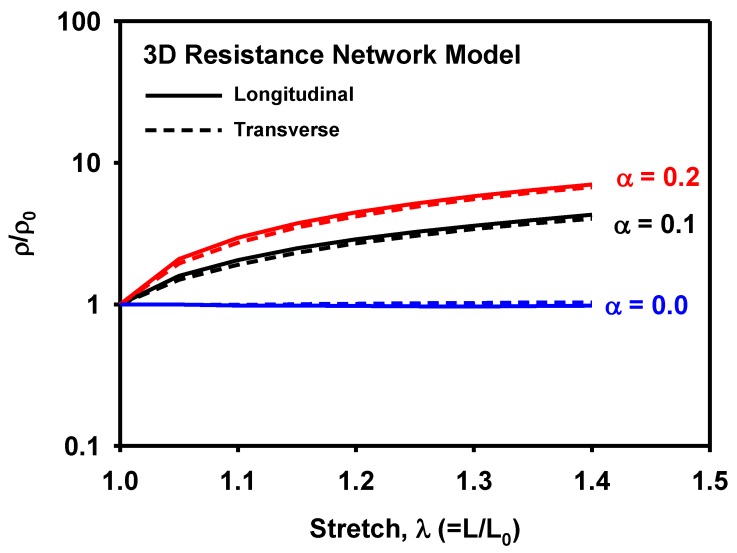
Variation of normalized resistivity with various values of α as calculated by the 3D resistance network model.

Figure 17 shows the effect of the parameter α on the resistivity evolution. If α increases or the CNTs are more affinely connected to the matrix, the resistivity increases. On the other hand, if α decreases or more slippage between CNTs and matrix occurs, the resistivity decreases. If α=0, no change of resistivity was predicted. This indicates that the resistivity of CNTs itself and the interface between CNTs and the matrix play a major role in the piezoresistivity of the CNT/TPU composites. It also explains why the piezoresistivity in the transverse direction agree with that in the longitudinal direction during the stretching in the longitudinal direction. 

**Figure 18:**
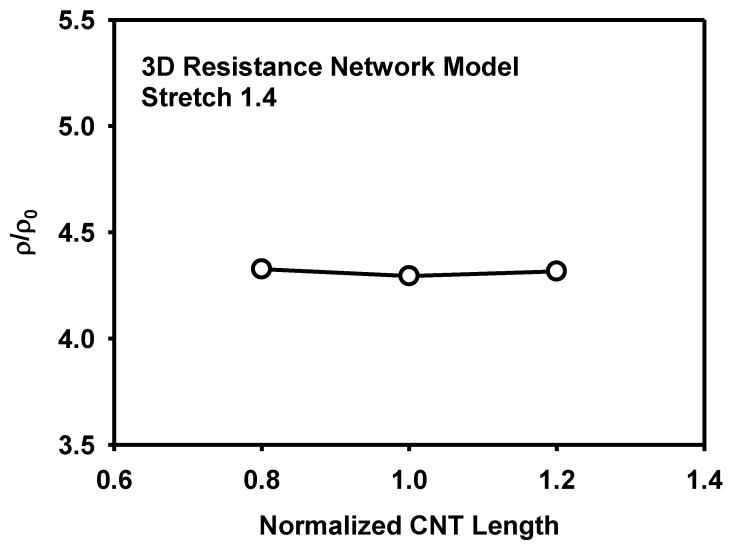
Effect of the CNT length on the resistivity as calculated by the 3D resistance network model.

**Figure 19:**
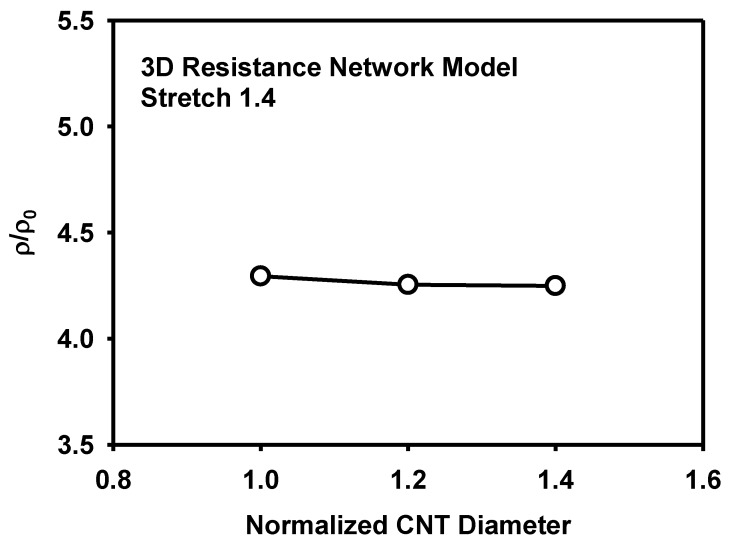
Effect of the CNT diameter on the resistivity as calculated by the 3D resistance network model.

**Figure 20:**
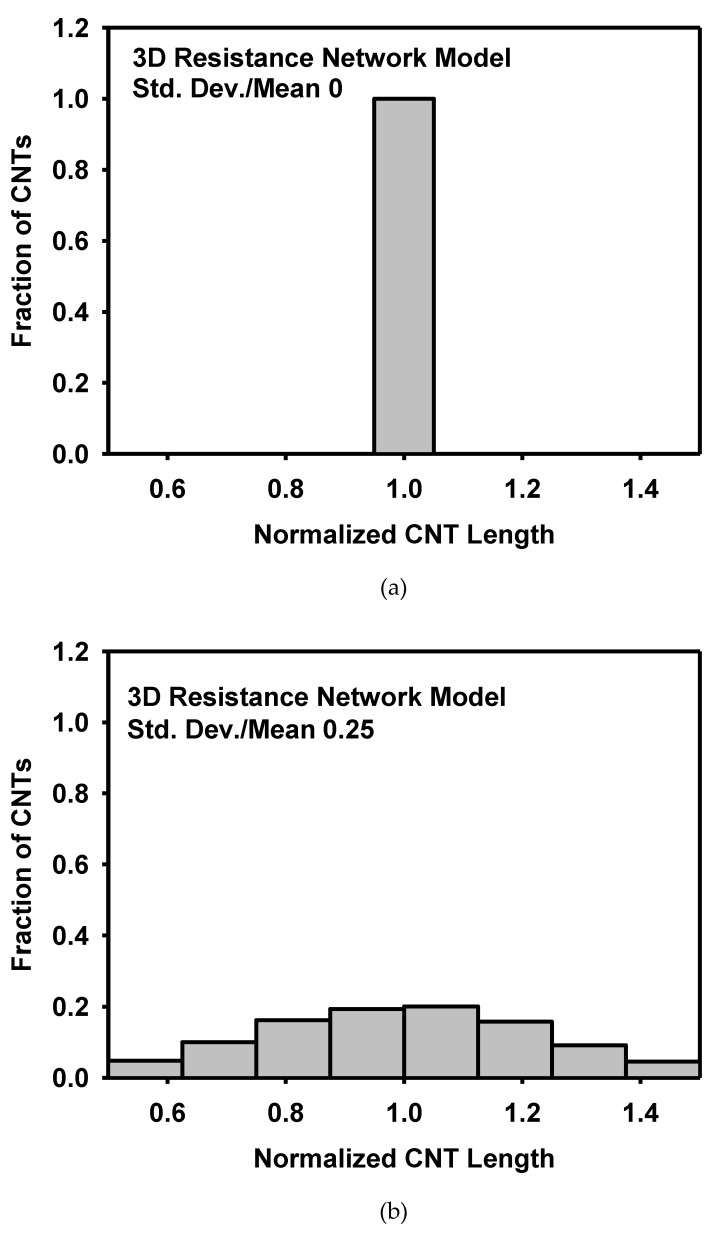

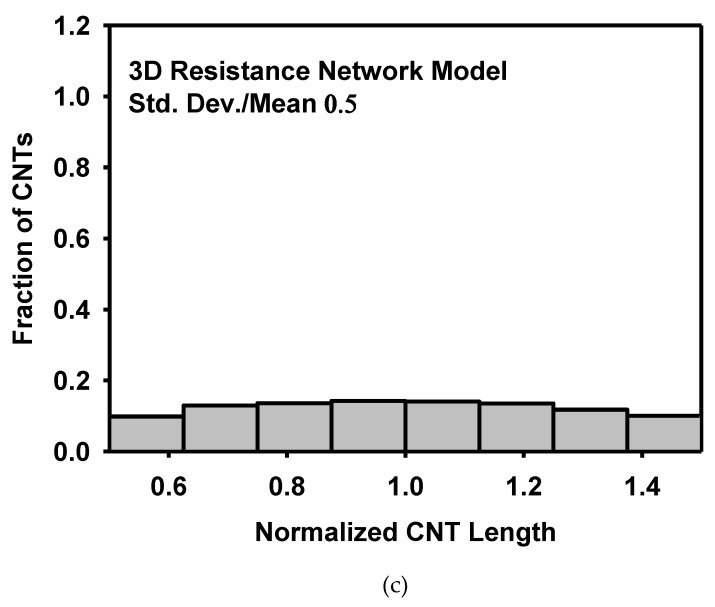
Three distributions of the CNT lengths used in the 3D resistance network model with the ratio of standard deviation to mean CNT length of (**a**) 0, (**b**) 0.25, and (**c**) 0.5.

**Figure 21:**
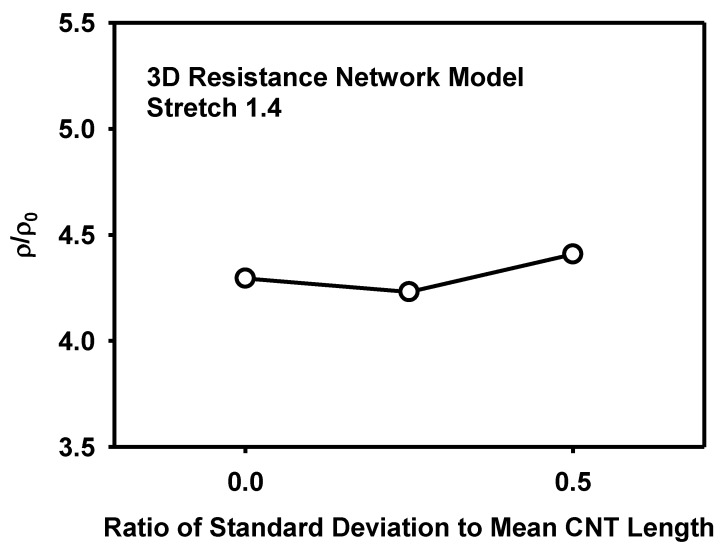
Effect of the CNT length distribution on the resistivity as calculated by the 3D resistance network model.

The effect of the CNT size and its distribution on the piezoresistivity is analysed as follows. Figure 18 shows the effect of the CNT length on the resistivity at the stretch of 1.4. It is known that the increase of CNT length decreases sensitivity or piezoresistivity [14]. However, the CNT length did not show significant difference in the present models at the given range of length variation and at the specific CNT content of 5 wt%. Figure 19 shows the effect of the CNT diameter on the resistivity at the stretch of 1.4. Though the amount is small, the increase of diameter lowers the resistivity, similarly to the effect of the CNT length. To analyse the effect of the size distribution on the piezoresistivity, 3D resistance network models with different distributions of CNT lengths were generated as shown in Figure 20. The CNT lengths were varied to have normal distributions with ratios of standard deviation to mean CNT length of 0.25 and 0.5. Though the effect of the size distribution is not large, the model with diverse CNT lengths showed higher piezoresistivity than the model with fixed CNT lengths in Figure 21. 

**Figure 22:**
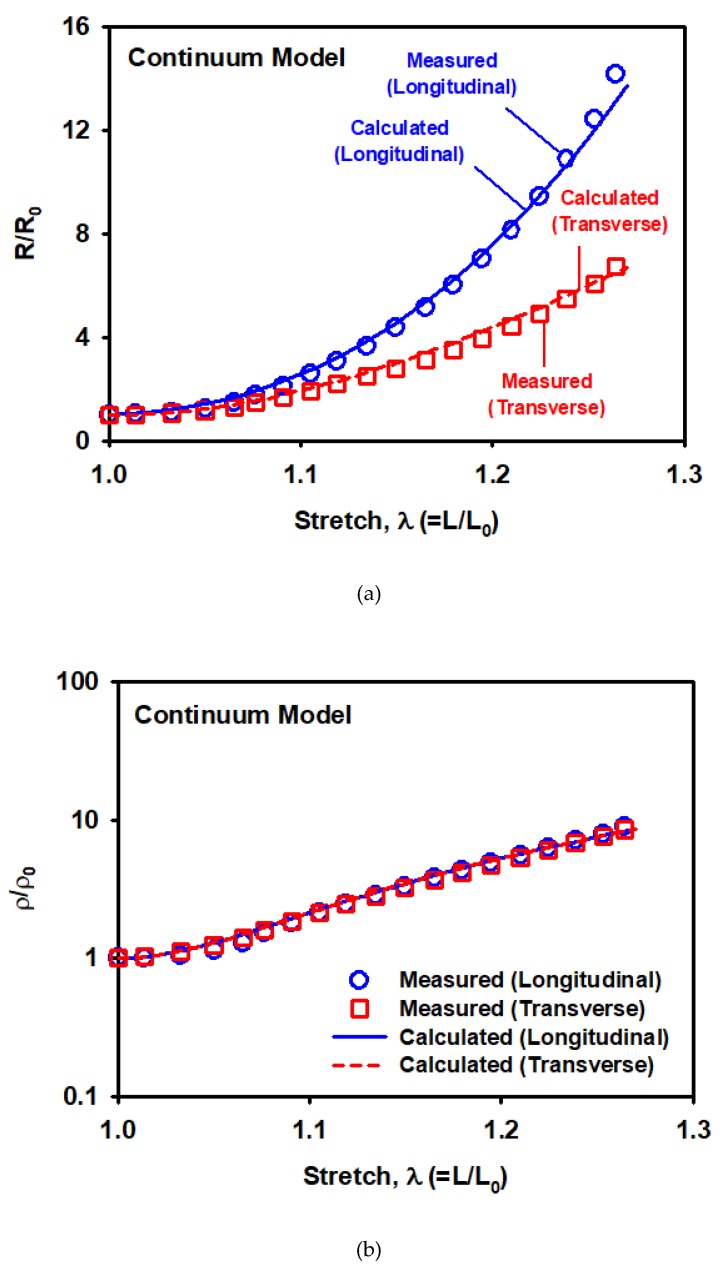
(**a**) Normalized resistance and (**b**) normalized resistivity predicted by the continuum resistivity model.

The resistivity curves were fitted using the continuum resistivity model, and material parameters are listed in Table 3. The model can describe the evolution of the resistivity in the longitudinal and transverse directions well, as shown in Figure 22. 

## 4. Conclusions

In this study, a MWCNT/TPU filament was developed by adding a conductive filler (MWCNTs) to fabricate sensors directly using FDM 3D printing. Since this material is based on a flexible TPU, it can be applied to various devices, including wearable sensors and equipment that require flexibility. The conductivity was analysed in terms of the MWCNT concentration. The percolation threshold value was verified with the sharp increase in conductivity at an MWCNT concentration of 3 wt%. 

The piezoresistivity of the MWCNT/TPU composite with an MWCNT concentration of 5 wt% was characterised by performing tensile tests using the 3D-printed specimens up to a 40% strain along the loading and transverse directions while simultaneously measuring the resistance. The results showed that the isotropy of resistivity was preserved during deformation. A 3D resistance network model was used to simulate the conduction phenomenon of the MWCNT/TPU composites. The piezoresistivity analysis using a 3D resistance network model also confirmed the isotropic evolution of resistivity. In a macro-scale approach, the continuum model based on the stretch function was proposed and had good consistency with the experimental data.

## Figures and Tables

**Table 1:** Printing conditions for specimens.

Parameter	Value
layer height (mm)	0.2
infill percentage (%)	100
infill angle offsets (°)	45
nozzle diameter (mm)	0.8
extrusion multiplier	1.2
heated build platform temperature (°C)	60
extruder temperature (°C)	230
printing speed (mm/s)	85

**Table 2:** Material parameters for 3D resistance network model.

Parameter	Value
α	0.1
diameter of CNT [nm]	5
length of CNT [nm]	400
number of CNTs	5691
conductivity of CNT [S/nm]	0.00001
contact resistance [Ω]	100
gauge factor of CNT	400

**Table 3:** Parameters of the continuum resistivity model.

Parameter	Value
$\rho_{0}$ [Ω·mm]	10,000
C	40
